# A synopsis of the Bee occurrence data of northern Tanzania

**DOI:** 10.3897/BDJ.9.e68190

**Published:** 2021-08-17

**Authors:** Julius V. Lasway, Neema R. Kinabo, Rudolf F. Mremi, Emanuel H. Martin, Oliver C. Nyakunga, John J. Sanya, Gration M. Rwegasira, Nicephor Lesio, Hulda Gideon, Alain Pauly, Connal Eardley, Marcell K. Peters, Andrew T. Peterson, Ingolf Steffan-Dewenter, Henry K. Njovu

**Affiliations:** 1 Department of Wildlife Management, College of African Wildlife Management, Mweka, P.O. Box 3031, Moshi, Tanzania Department of Wildlife Management, College of African Wildlife Management, Mweka, P.O. Box 3031 Moshi Tanzania; 2 Department of Animal Ecology and Tropical Biology, Biocenter, University of Würzburg, Am Hubland, 97074 Würzburg, Germany Department of Animal Ecology and Tropical Biology, Biocenter, University of Würzburg, Am Hubland 97074 Würzburg Germany; 3 Department of Wildlife Tourism, College of African Wildlife Management, Mweka, P.O. Box 3031, Moshi, Tanzania Department of Wildlife Tourism, College of African Wildlife Management, Mweka, P.O. Box 3031 Moshi Tanzania; 4 Department of Crop Science and Hortculture, Sokoine University of Agriculture, P.O. Box 3005, Morogoro, Tanzania Department of Crop Science and Hortculture, Sokoine University of Agriculture, P.O. Box 3005 Morogoro Tanzania; 5 Tanzania Wildlife Research Institute, P.O. Box 661, Arusha, Tanzania Tanzania Wildlife Research Institute, P.O. Box 661 Arusha Tanzania; 6 Tanzania Commission for Science and Technology (COSTECH), P.O. Box 4302, Ali Hassan Mwinyi Road, Dar es Salaam, Tanzania Tanzania Commission for Science and Technology (COSTECH), P.O. Box 4302, Ali Hassan Mwinyi Road Dar es Salaam Tanzania; 7 Royal Belgian Institute of Natural Sciences (RBINS), O.D. Taxonomy & Phylogeny, Rue Vautier 29, B-1000, Brussels, Belgium Royal Belgian Institute of Natural Sciences (RBINS), O.D. Taxonomy & Phylogeny, Rue Vautier 29, B-1000 Brussels Belgium; 8 Unit for Environmental Sciences and Management,, North-West University, Potchefstroom 2520, South Africa Unit for Environmental Sciences and Management, North-West University, Potchefstroom 2520 South Africa; 9 Department of Ecology and Evolutionary Biology, University of Kansas, Lawrence, KS, United States of America Department of Ecology and Evolutionary Biology, University of Kansas Lawrence, KS United States of America; 10 Wildlife Conservation Society of Tanzania, P.O. Box 70919, Dar Es Salaam, Tanzania Wildlife Conservation Society of Tanzania, P.O. Box 70919 Dar Es Salaam Tanzania

**Keywords:** agriculture, bee pollinator, distribution, disturbance gradient, grazing, species diversity, Tanzania

## Abstract

**Background:**

Bees (Hymenoptera: Apoidea: Anthophila) are the most important group of pollinators with about 20,507 known species worldwide. Despite the critical role of bees in providing pollination services, studies aiming at understanding which species are present across disturbance gradients are scarce. Limited taxononomic information for the existing and unidentified bee species in Tanzania make their conservation haphazard. Here, we present a dataset of bee species records obtained from a survey in nothern Tanzania i.e. Kilimanjaro, Arusha and Manyara regions. Our findings serve as baseline data necessary for understanding the diversity and distribution of bees in the northern parts of the country, which is a critical step in devising robust conservation and monitoring strategies for their populations.

**New information:**

In this paper, we present information on 45 bee species belonging to 20 genera and four families sampled using a combination of sweep-netting and pan trap methods. Most species (27, ~ 60%) belong to the family Halictidae followed by 16 species (35.5%) from the family Apidae. Megachilidae and Andrenidae were the least represented, each with only one species (2.2%). Additional species of Apidae and Megachilidae sampled during this survey are not yet published on Global Biodiversity Information Facility (GBIF), once they will be available on GBIF, they will be published in a subsequent paper. From a total of 953 occurrences, highest numbers were recorded in Kilimanjaro Region (n = 511), followed by Arusha (n = 410) and Manyara (n = 32), but this pattern reflects the sampling efforts of the research project rather than real bias in the distributions of bee species in northern Tanzania.

## Introduction

Bees (Hymenoptera: Apoidea: Anthophila) play an important ecological role in ecosystem. They serve a pollination role through mutualistic interactions with plants that in turn maintain the functionality of natural ecosystem, enhancing crop production and hence promoting human well being (Potts et al. 2016). Improved pollination service is essential for biodiversity conservation because plants act as primary producers in ecosystem. Nonetheless, they provide a vast array of ecosystem services: carbon sequestration, soil erosion prevention, nitrification and maintaining water tables, just to name a few. About 94% flowering plants reproduction depend on animal pollination in particular bee pollinators (Ollerton et al. 2011). Therefore, bees are considered as the most important pollinator of crops and wild plants as they can visit more than 90% of the leading 107 global crop types (Klein et al. 2007).

Taxonomic information of bee species in many parts of the world is poorly understood (Williams et al. 2001, Winfree 2010, Eardley et al. 2016) and Tanzania is no exception. The distribution and diversity of wild bee species in Tanzania is equivocal, given the lack of a countrywide bee catalogue and limited scientific studies. Tanzania is renowned for its unique biodiversity and high endemism (URT 2014). With a mainland area of 945,087 km^2^, lack of information on distribution and diversity of bee species poses a risky scenario, as unknown bee species may dissappear even before they are discovered and documented. On the other hand, decline of bee populations are increasingly becoming a global concern, a situation which jeopardizes provision of pollination sevices to both natural and agro-ecosystems (Westphal et al. 2008, Cameron et al. 2011, Koh et al. 2016, Potts et al. 2016, Tommasi et al. 2021). Nonetheless, knowledge of local bee fauna, including species present and their distribution, is worthy understanding and should be a conservation concern regardless of their importance in the agriculture sector. Research shows that land-use intensification, climate change, introduction of alien invasive species and pathogens are amongst the major driving factors for bee populations declines (Potts et al. 2016, Bartomeus and Dicks 2019). There is also lack of empirical data on synergistic interaction of such factors owing to their interconnection and complexity which impedes the management and conservation of wild bee pollinators (Westphal et al. 2008, Gemmill-Herren et al. 2014, Potts et al. 2016).

In recent years, a few studies have provided partial information on the ecology of bees in Tanzania (Classen et al. 2015, Classen et al. 2017, Classen et al. 2020). However, these studies focused on bee diversity using morphospecies, plant-bee interactions and body size trait along elevation gradients of Mt.Kilimanjaro. Additionally, some studies on bee species conducted in the country were confined to a specific taxon, for example, in the genus *Apis* (Mumbi et al. 2014) and tribe Meliponini (Hamisi 2019). To date, no studies have comprehensively compiled occurrence of bee species in Tanzania to understand their diversity and distribution. In 2017, the College of African Wildlife Management, Mweka (CAWM), in collaboration with local and international partners, developed a three-year Bee Pollinator Monitoring Project to bridge this information gap. On this account, this paper presents bee occurrence data of northern Tanzania (Kilimanjaro, Arusha and Manyara administrative regions) with reference to an online dataset shared to the wider scientific community throughhttps://doi.org/10.15468/hdcdf3 (Lasway et al. 2021). The result is a qualitative improvement in the availability of primary data on the bee species of this country.

## Project description

### Title

Bee - Pollinator Monitoring Project , Tanzania

### Personnel

The project is hosted at CAWM, Mweka Tanzania and is being implemented in collaboration with local and international partner institutions. Local institutions include Sokoine University of Agriculture (SUA), Tanzania Wildlife Research Institute (TAWIRI), Tanzania Commission for Science and Technology (COSTECH), Ministry of Agriculture - Tanzania, Tropical Pesticide Research Institute (TPRI) and National Museum of Tanzania (NMT). Partner institutions from outside Tanzania include the University of Würzburg (Germany), Agricultural Research Council ARC (South Africa), Royal Belgian Institute of Natural Sciences RBINS (Belgium), and the University of Kansas (USA).

**Goals**: The project's main goal was to determine the current distribution and status of bee pollinators in Tanzania. Other project objectives were:

- To strengthen the capacity of Tanzanians in the aspects of biodiversity informatics; plant-bee interactions; DNA-based and morphological identification techniques; and collection management;
- To develop and implement a standardized bee pollinator monitoring programme;
- To share data on bee species, abundance and their interactions with plants via dedicated databases, such as Global Biodiversity Information Facility (GBIF), Tanzania Biodiversity Information Facility (TanBIF) and African Pollinator Initiative (API);
- To disseminate results to the scientific community through peer-reviewed publications and conference presentations; and
- To raise awareness of the general public on the importance of bee pollinators through various media.

### Funding

The project is financed by the JRS Biodiversity Foundation, USA.

## Sampling methods

### Study extent

The study was carried out in a set of study sites established in agricultural (transformed), grazing (degraded) and natural savannah (conserved) lands to represent different land-use categories as presented in Table 1. Agriculture intensity was measured, based on magnitude of land use intensification, i.e. moderately intensive agriculture habitat was mainly characterized by smallholder farms with field sizes of less than 1 ha with mixed crops, such as maize, beans and sunflower. It is also characterized by moderate use of agricultural machines and agrochemicals, while intensive agriculture was characterized by monoculture farms. In this habitat, there is a high use of heavy agricultural machines and agricultural inputs (i.e. pesticides and chemical fertilizers). Nonetheless, grazing intensity was measured, based on the visual inspection of on-site signs of obvious grazing like shortened tufts of grass, presence or absence of livestock footprints and by calculating the distance between study sites to bomas (livestock enclosures and living grounds of families holding large herds of livestock) using remote sensing and GIS techniques. Study sites with signs of intensive grazing activity were very near to bomas (average distance 0.09 ± 0.05 (SD) km while study sites with moderate livestock grazing intensity were at a far distance to bomas (i.e. average distance 25.3 ± 27.6 km (SD).

### Sampling description

Data were collected in 40 study sites distributed along savannah, grazing and agriculture gradients in the three regions. A paired patch study design (i.e. sampling plots were positioned in two contrasting habitats within each study site) was used to minimize spatial autocorrelation. In each study site, two 50 x 50 m sampling plots were positioned and spaced at least 150 m apart. The coordinates of the plots were recorded at the mid-point between the paired plots. Bee data collection involved a combination of standardized pan trapping and random walk methods. These techniques have successfully been used for sampling bee species in northern Tanzania (Classen et al. 2015, Classen et al. 2017, Classen et al. 2020) and in other parts of the world (e.g. Noyes 1989, Stephen and Rao 2007, Westphal et al. 2008, Yi et al. 2012, Spafford and Lortie 2013). In each plot, four clusters of UV-Reflecting pan traps (each with yellow, white and blue) were installed and left in the field to collect bees for 48 hours. Two of the clusters were installed using a 120 cm pole to increase the chances of collecting bees foraging on shrubs and the other two were installed using a 35 cm pole to capture bees foraging on herbaceous plants. In each of three quota water-filled pan traps, a drop of scentless colourless liquid soap was added to break the surface tension and prevent bees from escaping. The total sampling effort for this technique summed to 1,152 hours per site. For the standardized random walk, two researchers actively collected bees for two hours within each sampling plot using sweep nets. This method summed to a sampling effort of four man-hours per study site.

### Quality control

Controlling data: For each of the study sites, we recorded the habitat type, GPS coordinates and elevation (metres above sea level, m a.s.l.). The coordinates and elevation of localities were derived from a hand-held Garmin GPS (Model: GPSMAP64s; resolution ± 3 m; Garmin Ltd, Taiwan). In addition, for each study site, information on weather parameters (temperature and precipitation) and forage resources were recorded. The specimens collected were preserved in 70% ethanol before being mounted and identified by afro-tropical bee taxonomists (Alain Pauly and Connal Eardley). Bees were identified following the nomenclatural system of Michener 2007 "The Bees of the World, Second Edition" with the exception of the family Halictidae that followed Pauly 1990 and Pauly 1999. Both Michener (2007) and Halictidae taxonomic publications contain keys, diagnosis and descriptions of bees. The reference collections for identified bee species are available at the CAWM, Mweka.

## Geographic coverage

### Description

**Figure 1.**
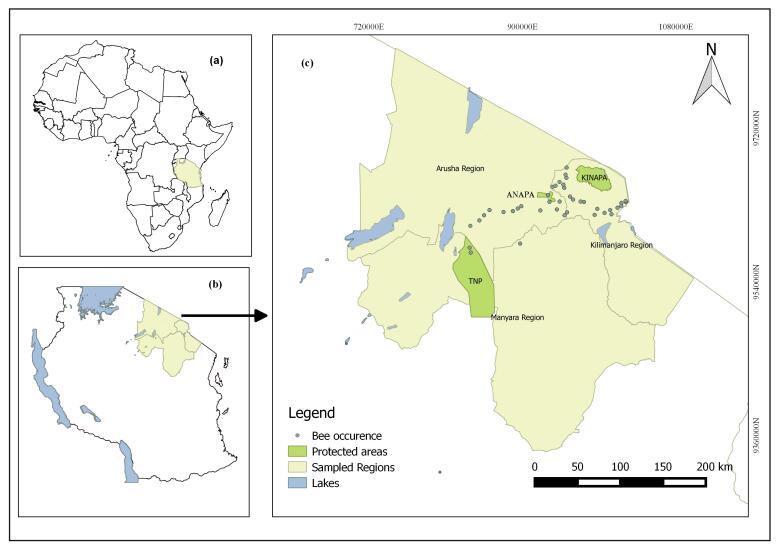
Map of the study area showing study sites. **(a)** Location of Tanzania (pale yellow background) on the map of Africa; **(b)** Location of the study area (pale yellow background) in Tanzania; **(c)** Enlarged map of the study area showing sampling sites (grey dots) in northern Tanzania i.e Kilimanjaro, Arusha and Manyara regions.

The study was conducted in the northern part of Tanzania i.e. Kilimanjaro, Arusha and Manyara regions (Fig. 1). The study regions are located between latitude 3°30’ S and 4°45’ S and longitude 4°30' E and 5°45’ E. The study regions have two rainy seasons: a long rainy season from March to May and a short rainy season in November and December. Average annual rainfall ranges geographically between 1300 mm and 2400 mm. Annual mean maximum temperature (hottest season) is 25.4°C between July and September and minimum temperature (cold season) is 12.8°C between May and June.

### Coordinates

3°30’ S and 4°45’ S Latitiude and Latitude; and 4°30' E and 5°45’ E Longitude Longitude.

## Taxonomic coverage

### Description

This data paper describes a total of 953 occurrences for bee species representing four families, 20 genera and 45 species (Table 2), amongst 20,507 species that have been described worldwide (Ascher and Pickering 2020). Seven families of bee species (Andrenidae, Halictidae, Apidae, Melittidae, Colletidae, Megachilidae and Stenotridae) are currently recognized globally (Michener 2007), though only four (Andrenidae, Apidae, Halictidae and Megachilidae) have been recorded in this study. In this sample, seven species (*Apismellifera* (Linnaeus, 1758), *Macrogaleacandida* (Smith, 1879), *Lasioglossumbowkeri* (Cockerell, 1920), *L.rubritarse* (Cockerell, 1937), *L.transvaalense* (Cameron & Cockerell, 1937), *Seladoniafoana* (Vachal, 1899) and *S.hotoni* (Vachal, 1903) are reported to occur across all land-use types: agricultural (transformation), grazing (degradation) and natural savannah (conservation), whereas other species are found in a subset of land-use types (Table 3).

The Halictidae was richest in species, with 27 species, followed by Apidae with 16 species. Two families (Andrenidae and Megachilidae) were represented by single species: *Andrenanotophila* (Cockerell, 1933) and *Lithurguspullatus* (Vachal, 1903), respectively (Table 3). Greater numbers of records from Kilimanjaro (511 occurrences), compared to Arusha (410 occurrences) and Manyara (32 occurrences) is attributed to more sample plots in the region and not fewer bee species in Arusha or Manyara regions.

## Temporal coverage

**Data range:** 2018-8-06 – 2018-12-21.

### Notes

Bees were collected intermittently between August and December 2018. Two study sites were visited per day for data collection using pan trap and sweep-net methods. Pan traps were left in the field to collect bees for 48 hours before they were emptied and moved to the next study site. Additionally, sweep-netting was used to collect bee species actively for two hours per study site, excluding handling and processing time. Data collection by handnet was conducted when bees were most active in the morning between 9:00 and 11:00 am.

## Usage licence

### Usage licence

Creative Commons Public Domain Waiver (CC-Zero)

### IP rights notes

These data can be freely used, provided their source is cited.

## Data resources

### Data package title

Occurrence of bees along grazing and agricultural gradients in northern Tanzania

### Resource link

https://doi.org/10.15468/hdcdf3

### Number of data sets

1

### Data set 1.

#### Data set name

Occurrence of bees along grazing and agricultural gradients in northern Tanzania

#### Data format

Darwin Core Archive

#### Number of columns

21

#### Download URL

https://bit.ly/32tklEA

#### Description

The data were prepared following DARWIN CORE format

**Data set 1.** 

Column label	Column description
institutionCode	The acronym in use by the institution having custody of the information referred to in the record.
basisOfRecord	The specific nature of the data record.
occurrenceID	The Globally Unique Identifier number for the record.
individualCount	The number of individuals that were recorded
habitat	A category or description of the habitat in which the Event occurred.
countryCode	The standard code for the country in which the Location occurs
decimalLatitude	The verbatim original latitude of the Location.
decimalLongitude	The verbatim original longitude of the Location.
scientificName	The full scientific name including the genus name and the lowest level of taxonomic rank with the authority.
kingdom	The full scientific name of the kingdom in which the taxon is classified
eventDate	The date or date interval during which the occurrence record was collected.
geodeticDatum	The coordinate system and set of reference points upon which the geographic coordinates are based.
coordinateUncertaintyInMetres	The horizontal distance from the given decimalLatitude and decimalLongitude in metres, describing the smallest circle containing the whole of the Location.
organismQuantity	A number or enumeration value for the quantity of organisms.
organismQuantityType	The type of quantification system used for the quantity of organisms
samplingProtocol	The description of the method used during sampling
taxonRank	The taxonomic rank of the most specific name in the scientificName.
scientificNameAuthorship	The authorship information for the scientificName formatted according to the conventions of the applicable nomenclaturalCode.
ScientificName	The full scientific name of a taxon.
acceptedNameUsage	The full name, with authorship and date information, if known, of the currently valid or accepted taxon.
taxonomicStatus	The status of the use of the scientificName as a label for a taxon

## Figures and Tables

**Table 1.** The regional study sites location

Study site	Study site ID	Latitude	Longitude	Elevation (m a.s.l.)	Region
Miwaleni	IA1	-3.4233	37.4604	702	Kilimanjaro
Mjohoroni	IA2	-3.3813	37.3836	764	Kilimanjaro
Kahe	IA3	-3.4451	37.3564	741	Kilimanjaro
Lambo estate	IA4	-3.3095	37.2436	1007	Kilimanjaro
Bomang'ombe	IA5	-3.2834	37.1292	1036	Kilimanjaro
West Kilimanjaro	IA6	-3.0205	37.0488	1497	Kilimanjaro
West Kilimanjaro	IA7	-2.9461	37.0597	1708	Kilimanjaro
Kikatiti	IA8	-3.3892	36.9592	1047	Arusha
NARCO	IG1	-3.098	36.9852	1359	Kilimanjaro
Lekrumuni	IG2	-3.1378	36.9434	1404	Kilimanjaro
Lekrumuni	IG3	-3.1508	36.9115	1391	Kilimanjaro
KIA	IG4	-3.4504	37.0394	890	Kilimanjaro
Meserani	IG5	-3.4079	36.4956	1330	Arusha
Arkatani	IG6	-3.418	36.3967	1327	Arusha
Bwawani	IG7	-3.4531	36.1923	1314	Arusha
Makuyuni juu	IG8	-3.5081	36.1431	1227	Arusha
Njia panda	MIA1	-3.3932	37.5191	847	Kilimanjaro
Njia panda	MIA2	-3.4375	37.5334	759	Kilimanjaro
Kibo estate	MIA3	-3.3044	37.2078	1025	Kilimanjaro
Donyo Moru	MIA4	-3.2526	37.0964	1101	Kilimanjaro
New Molomo farm	MIA5	-3.1602	37.0356	1376	Kilimanjaro
King'ori	MIA6	-3.3058	36.9875	1167	Arusha
Kisongo	MIA7	-3.3809	36.5465	1368	Arusha
Nanja	MIA8	-3.3981	36.2493	1478	Arusha
Challa	MIG1	-3.3162	37.6383	1137	Kilimanjaro
Challa	MIG2	-3.3475	37.6357	1023	Kilimanjaro
Holili	MIG3	-3.3682	37.5968	940	Kilimanjaro
Dachkona	MIG4	-3.1259	37.0264	1380	Kilimanjaro
Mwangaza	MIG5	-3.0544	37.0575	1532	Kilimanjaro
KIA	MIG6	-3.4187	37.0668	900	Kilimanjaro
NelsonMandela	MIG7	-3.4002	36.7848	1216	Arusha
UN	MIG8	-3.3562	36.5838	1441	Arusha
Challa	SAV1	-3.3091	37.685	945	Kilimanjaro
Challa	SAV2	-3.2957	37.6817	954	Kilimanjaro
ANAPA	SAV3	-3.2372	36.8663	1406	Arusha
ANAPA	SAV4	-3.309	36.8803	1576	Arusha
Manyara ranch	SAV5	-3.5657	36.0478	1065	Manyara
TARNAPA	SAV6	-3.7476	41.9738	1031	Manyara
TARNAPA	SAV7	-3.7944	36.0406	1071	Manyara
TARNAPA	SAV8	-3.846	36.0525	1073	Manyara

IA = Intensive Agriculture; IG = Intensive Grazing; MIA = Mid-Intensive Agriculture; MIG = Mid-Intensive Grazing; SAV= Savannah

**Table 2.** Summary of bee occurrence records from northern Tanzania by family.

Class	Order	Family	No. of genera recorded	No. of species recorded	No. of individuals recorded
Insecta	Hymenoptera	Andrenidae	1	1	1
		Apidae	9	16	570
		Halictidae	9	27	352
		Megachilidae	1	1	30
Total			20	45	953

**Table 3.** Species list of bee data records from northen Tanzania.

Family	Genera	Scientific name and authorship	Land-use type
Andrenidae	* Andrena *	*Andrenanotophila* (Cockerell, 1933)	Savannah habitat in Arusha region.
Apidae	* Apis *	*Apismellifera* (Linnaeus, 1758)	Savannah, intensive agriculture, intensive grazing, mid-intensive agriculture and mid-intensive grazing habitat in Arusha and Kilimanjaro regions.
Apidae	* Eucara *	*Eucaramacrognatha* (Gerstaecker, 1870)	Mid-intensive agriculture and mid-intensive grazing habitat in Kilimanjaro region.
Apidae	* Hypotrigona *	*Hypotrigonagribodoi* (Magretti, 1884)	Mid-intensive agriculture and mid-intensive grazing habitat in Kilimanjaro region.
Apidae	* Liotrigona *	*Liotrigonabottegoi* (Magretti, 1895)	Savannah habitat in Kilimanjaro region.
Apidae	* Macrogalea *	*Macrogaleacandida* (Smith, 1879)	Savannah, intensive agriculture, intensive grazing, mid-intensive agriculture and mid-intensive grazing habitat in Arusha and Kilimanjaro regions.
Apidae	* Meliponula *	*Meliponulaferruginea* (Lepeletier, 1836)	Intensive agriculture habitat in Arusha region.
Apidae	* Meliponula *	*Meliponulatogoensis* (Stadelmann)	Savannah and mid-intensive grazing habitat in Arusha and Kilimanjaro regions.
Apidae	* Pleibena *	*Plebeinaarmata* (Magretti, 1895)	Mid-intensive grazing habitat in Kilimanjaro region.
Apidae	* Schwarzia *	*Schwarziaemmae* (Eardley, 2009)	Intensive agriculture habitat in Kilimanjaro region.
Apidae	* Xylocopa *	*Xylocopacaffra* (Linnaeus, 1767)	Intensive agriculture and mid-intensive grazing habitat in Kilimanjaro region.
Apidae	* Xylocopa *	*Xylocopaerythrina* (Gribodo, 1894)	Intensive grazing habitat in Kilimanjaro region.
Apidae	* Xylocopa *	*Xylocopaflavicollis* (DeGeer,1778)	Intensive agriculture, mid-intensive agriculture and mid-intensive grazing habitat in Arusha and Kilimanjaro regions.
Apidae	* Xylocopa *	*Xylocopaflavorufa* (DeGeer, 1778)	Intensive agriculture habitat in Kilimanjaro region.
Apidae	* Xylocopa *	*Xylocopainconstans* (Smith,1874)	Intensive agriculture, intensive grazing, mid-intensive agriculture habitat in Arusha and Kilimanjaro regions.
Apidae	* Xylocopa *	*Xylocopanigrita* (Fabricius, 1775)	Intensive agriculture habitat in Kilimanjaro region.
Apidae	* Xylocopa *	*Xylocopasomalica* (Magretti, 1895)	Intensive agriculture, intensive grazing, mid-intensive agriculture habitat in Arusha and Kilimanjaro regions.
Halictidae	* Acunomia *	*Acunomiatheryi* (Gribodo,1894)	Intensive agriculture, mid-intensive agriculture habitat in Arusha and Kilimanjaro regions.
Halictidae	* Crocisaspidia *	*Crocisaspidiachandleri* (Ashmead,1899)	Mid-intensive agriculture habitat in Kilimanjaro region.
Halictidae	* Crocisaspidia *	*Crocisaspidiaforbesii* (Kirby, 1900)	Intensive grazing habitat in Kilimanjaro region.
Halictidae	* Lasioglossum *	*Lasioglossumacuiferum* (Cockerell, 1935)	Savannah, intensive grazing, mid-intensive agriculture and mid-intensive grazing habitat in Manyara, Arusha and Kilimanjaro regions.
Halictidae	* Lasioglossum *	*Lasioglossumatricrum* (Vachal, 1903)	Intensive agriculture, mid-intensive grazing habitat in Arusha and Kilimanjaro regions.
Halictidae	* Lasioglossum *	*Lasioglossumbellulum* (Vachal, 1910)	Intensive agriculture, intensive grazing, mid-intensive agriculture and mid-intensive grazing habitat in Arusha and Kilimanjaro regions.
Halictidae	* Lasioglossum *	*Lasioglossumbowkeri* (Cockerell, 1920)	Savannah, intensive agriculture, intensive grazing, mid-intensive agriculture and mid-intensive grazing habitat in Arusha and Kilimanjaro regions.
Halictidae	* Lasioglossum *	*Lasioglossumdeceptum* (Smith, 1853)	Intensive agriculture, mid-intensive agriculture habitat in Arusha and Kilimanjaro regions.
Halictidae	* Lasioglossum *	*Lasioglossumhancocki* (Cockerell, 1945)	Intensive agriculture, intensive grazing, mid-intensive agriculture and mid-intensive grazing habitat in Arusha and Kilimanjaro regions.
Halictidae	* Lasioglossum *	*Lasioglossummatopiense* (Cockerell, 1940)	Savannah, intensive grazing, mid-intensive agriculture and mid-intensive grazing habitat in Manyara, Arusha and Kilimanjaro regions.
Halictidae	* Lasioglossum *	*Lasioglossumrubritarse* (Cockerell, 1937)	Savannah, intensive agriculture, intensive grazing, mid-intensive agriculture and mid-intensive grazing habitat in Arusha and Kilimanjaro regions.
Halictidae	* Lasioglossum *	*Lasioglossumscobe* (Vachal, 1903)	Intensive agriculture, mid-intensive agriculture habitat in Arusha and Kilimanjaro regions.
Halictidae	* Lasioglossum *	*Lasioglossumtransvaalense* (Cameron&Cockerell, 1937)	Savannah, intensive agriculture, intensive grazing, mid-intensive agriculture and mid-intensive grazing habitat in Manyara, Arusha and Kilimanjaro regions.
Halictidae	* Macronomia *	*Macronomiaarmatula* (Dalla Torre, 1896)	Savannah habitat in Manyara region.
Halictidae	* Nubenomia *	*Nubenomiareichardia* (Strand, 1911)	Savannah, intensive agriculture, mid-intensive agriculture habitat in Manyara and Kilimanjaro regions.
Halictidae	* Pachynomia *	*Pachynomiaflavicarpa* (Vachal, 1903)	Mid-intensive grazing habitat in Kilimanjaro region.
Halictidae	* Patellapis *	*Patellapisitigiensis* (Kuhlmann & Pauly, 2010)	Intensive agriculture habitat in Kilimanjaro region.
Halictidae	* Pseudapis *	*Pseudapispandeana* (Strand, 1914)	Mid-intensive agriculture and mid-intensive grazing habitat in Arusha and Kilimanjaro regions.
Halictidae	* Pseudapis *	*Pseudapisusambarae* (Pauly, 1990)	Mid-intensive agriculture in Kilimanjaro region.
Halictidae	* Seladonia *	*Seladoniaafricana* (Friese, 1909)	Intensive agriculture, mid-intensive agriculture habitat in Kilimanjaro region.
Halictidae	* Seladonia *	*Seladoniafoana* (Vachal, 1899)	Savannah, intensive agriculture, intensive grazing, mid-intensive agriculture and mid-intensive grazing habitat in Arusha and Kilimanjaro regions.
Halictidae	* Seladonia *	*Seladoniahotoni* (Vachal, 1903)	Savannah, intensive agriculture, intensive grazing, mid-intensive agriculture and mid-intensive grazing habitat in Manyara, Arusha and Kilimanjaro regions.
Halictidae	* Seladonia *	*Seladonialucidipennis* (Smith, 1853)	Mid-intensive agriculture habitat in Kilimanjaro region.
Halictidae	* Steganomus *	*Steganomusjunodi* (Gribodo, 1895)	Savannah, mid-intensive agriculture and mid-intensive grazing habitat in Manyara, Arusha and Kilimanjaro regions.
Halictidae	* Trinomia *	*Trinomiacirrita* (Vachal, 1903)	Savannah, intensive agriculture,mid-intensive grazing habitat in Manyara and Arusha regions.
Halictidae	* Zonalictus *	*Zonalictuskabetensis* (Cockerell, 1937)	Savannah, intensive agriculture habitat in Arusha and Kilimanjaro regions.
Halictidae	* Zonalictus *	*Zonalictuskivuicola* (Cockerell, 1937)	Savannah, mid-intensive agriculture habitat in Arusha and Kilimanjaro regions.
Megachilidae	* Lithurgus *	*Lithurguspullatus* (Vachal, 1903)	Savannah, intensive agriculture, mid-intensive agriculture and mid-intensive grazing habitat in Arusha and Kilimanjaro regions.
